# Metacognition in Cardiac Patients With Anxiety and Depression: Psychometric Performance of the Metacognitions Questionnaire 30 (MCQ-30)

**DOI:** 10.3389/fpsyg.2020.01064

**Published:** 2020-05-26

**Authors:** Cintia L. Faija, David Reeves, Calvin Heal, Adrian Wells

**Affiliations:** ^1^Division of Nursing, Midwifery and Social Work, Faculty of Biology, Medicine and Health, Manchester Academic Health Science Centre, The University of Manchester, Manchester, United Kingdom; ^2^National Institute of Health Research (NIHR) School for Primary Care Research, Manchester Academic Health Science Centre, The University of Manchester, Manchester, United Kingdom; ^3^Faculty of Biology, Centre for Biostatistics, Medicine and Health, Manchester Academic Health Science Centre, The University of Manchester, Manchester, United Kingdom; ^4^Greater Manchester Mental Health National Health Service (NHS) Foundation Trust, Manchester, United Kingdom; ^5^Faculty of Biology, Medicine and Health, School of Psychological Sciences, Manchester Academic Health Science Centre, The University of Manchester, Manchester, United Kingdom

**Keywords:** cardiac patients, metacognition, metacognitive therapy, anxiety, depression, psychometrics

## Abstract

The evaluation of effective psychological therapies for anxiety and depression in cardiac patients is a priority, and progress in this area depends on the suitability and validity of measures. Metacognitive Therapy is a treatment with established efficacy in mental health settings. It postulates that anxiety and depression are caused by dysfunctional metacognitions, such as those assessed with the Metacognitions Questionnaire 30 (MCQ-30), which impair effective regulation of repetitive negative thinking patterns. The aim of this study was to examine the psychometric properties of the MCQ-30 in a cardiac sample. A sample of 440 cardiac patients with co-morbid anxiety and/or depression symptoms completed the MCQ-30 and the Hospital Anxiety and Depression Scale. Confirmatory factor analysis (CFA) was used to test established factor structures of the MCQ-30: a correlated five-factor model and a bi-factor model. The five-factor model just failed to meet our minimum criteria for an acceptable fit on Comparative Fit Index (CFI) = 0.892 vs. criterion of ≥ 0.9; but was acceptable on the Root Mean Square Error of Approximation (RMSEA) = 0.061 vs. ≤ 0.08; whereas the bi-factor model just met those criteria (CFI = 0.913; RMSEA = 0.056). These findings suggest that the bi-factor solution may carry additional information beyond the five subscale scores alone. However, such a model needs to be evaluated further before widespread adoption could be recommended. Meantime we recommend cautious continued use of the five-factor model. Structural issues aside, all five subscales demonstrated good internal consistency (Cronbach alphas > 0.7) and similar relationships to HADS scores as in other patient populations. The MCQ-30 accounted for additional variance in anxiety and depression after controlling for age and gender.

## Introduction

Anxiety and depression are common among patients with heart disease (Chaddha et al., 2016; Kosela et al., 2016; Chauvet-Gelinier and Bonin, 2017) and have been associated with adverse outcomes such as increased risk of mortality, poorer quality of life, and greater health care use (Palacios et al., 2018). The European Association of Cardiovascular Prevention and Rehabilitation reported that nearly 50% of patients attending cardiac rehabilitation services experience clinical levels of anxiety and/or depression (Pogosova et al., 2015) and effectiveness of current psychological interventions have been shown to be limited, i.e., non-significant improvements in reducing anxiety and depression (Jiang et al., 2018) or have only small effect sizes (Richards et al., 2017). Addressing psychological needs in this population could improve outcomes and reduce health care burden. Metacognitive therapy (MCT) (Wells, 2009) is a psychological treatment with established efficacy in mental health settings and is extending its evidence-base in patients with cardiac illnesses (Wells et al., 2018a, b).

MCT is based on an information processing model (Self-Regulatory Executive Function; S-REF) which postulates that metacognition plays a key role in the development and maintenance of anxiety and depression by causing perseverative negative thinking styles (Wells and Matthews, 1994, 1996). Metacognition is defined as the knowledge (beliefs) and cognitive processes involved in regulation and appraisal of thinking (Flavell, 1979). Two broad categories of metacognitive beliefs are distinguished in MCT: positive metacognitive beliefs, concerned with the advantages of worry, rumination and paying attention to threat; and negative metacognitive beliefs, focused on the concept that worrying/rumination is uncontrollable and/or dangerous (Wells, 2009). These metacognitions are thought to lead to a persistence of negative thinking in response to stress because they bias mental control in a way that undermines effective self-regulation (Wells, 2009).

Systematic reviews and meta-analyses provide robust evidence of positive relationships between metacognitions, anxiety and depression across mental health disorders, consistent with central predictions of the S-REF model (Rochat et al., 2017; Sun et al., 2017; Normann and Morina, 2018). But fewer studies have examined these relationships in physical health. Those that have show positive relationships between metacognitions and anxiety/depression in cancer patients (Cook et al., 2015), Parkinson’s disease (Allott et al., 2005), chronic fatigue (Maher-Edwards et al., 2012), epilepsy (Fisher et al., 2016), and cardiac patients (Anderson et al., 2019). Further testing of a metacognitive approach in the area of emotional distress in physical health depends on the suitability and validity of measures of metacognitions in this context. A limitation of existing research in the physical health area is evident in the paucity of studies that have confirmed the latent structure and reliability of the main measure of metacognitions used, the metacognitions questionnaire 30 (MCQ-30, Wells and Cartwright-Hatton, 2004). To support future research and to assess the impact of psychological treatment on metacognition in cardiac patients it is therefore mandatory to explore the structure, validity and reliability of the MCQ-30 to aid in the interpretation of findings.

### Study Aim

The present study was set out to test the construct validity and reliability of the MCQ-30 in a sample of patients undergoing cardiac rehabilitation experiencing mild to severe symptoms of anxiety and/or depression. Previous studies in different samples (e.g., non-clinical samples, psychiatric samples, cancer, and epilepsy), have proposed a correlated five-factor model (Wells and Cartwright-Hatton, 2004; Spada et al., 2008; Ramos-Cejudo et al., 2013; Cook et al., 2014), but a recent study conducted in a non-clinical sample provided initial evidence of a bi-factor model of the MCQ-30 (for a detailed explanation of the differences between a five-factor model and a bi-factor model of the MCQ-30 see Fergus and Bardeen, 2019). The bi-factor model consisted of the same five factors with the addition of a general factor contributing to all the individual items. However, none of these previous studies were conducted in a sample of cardiac patients. We cannot be certain that the structure of the MCQ-30 is retained in cardiac patients and this requires confirmation. For example, it is conceivable that experiencing cardiac illness may change the endorsement of beliefs about the usefulness or dangerousness of worry. Therefore, we investigated the applicability of both factor models of the MCQ-30 in this population, but because this is the first study exploring the psychometric properties of the MCQ-30 in cardiac patients, we also aimed to explore if an alternative factor structure might prove a better fit to the data. Our secondary aims were: (i) to explore associations between the MCQ-30 and anxiety and depression; and (ii) to examine which specific MCQ-30 subscales uniquely explained anxiety and depression symptoms.

## Materials and Methods

### Ethics Statement

This study draws on data collected under a 5 years programme of research funded by the National Institute for Health Research (NIHR) and sponsored by Greater Manchester Mental Health NHS Foundation Trust. The programme is called PATHWAY. Ethical approval was granted by the NHS Research Ethics Committee, United Kingdom. The Group-MCT Trial (Wells et al., 2018a) received ethical approval from Preston Research Ethics Committee (Ref: 15/NW/0163) and the Home-based MCT Feasibility Trial (Wells et al., 2018b) received ethical approval from the North West – Greater Manchester West Research Ethics Committee (Ref: 16/NW/0786).

### Participants and Procedure

Participants were recruited from seven UK National Health Service (NHS) sites delivering cardiac rehabilitation located in the Northwest of England. Patients referred to cardiac rehabilitation services were invited to take part in the programme if they met the following inclusion criteria: (i) score ≥ 8 on the depression and/or anxiety subscale of the HADS (Zigmond and Snaith, 1983); (ii) minimum of 18 years old; and (iii) competent level of English. Participants were excluded if they met any of the following: (i) cognitive impairment precluding informed consent or ability to participate; (ii) acute suicidality; (iii) active psychotic disorder; (iv) current drug/alcohol abuse; (v) concurrent psychological intervention for emotional distress that is not part of usual care; (vi) antidepressant or anxiolytic medications initiated 8 weeks prior to consent; and (vii) life expectancy of <12 months.

Patients meeting the inclusion criteria were identified by cardiac rehabilitation staff at NHS sites. Eligible and interested patients received an invitation and a patient information sheet. Patients willing to take part were asked to provide written informed consent and were then asked to complete measures at three different time-points (baseline, 4 and 12 months follow up). Data used in the present study include baseline measures only (before receiving any treatment). This is because the PATHWAY-Programme involved the delivery of a psychological intervention to half of the sample, and all participants received treatment as usual (cardiac rehabilitation programme) and we did not want responses on the MCQ-30 to be influenced by any intervention.

### Measures

#### Metacognitions Questionnaire 30 (MCQ-30; Wells and Cartwright-Hatton, 2004)

The MCQ-30 is a self-report measure comprised of 30 items. The items are grouped into five dimensions of metacognitive beliefs, each consisting of six items: (i) Cognitive Confidence (e.g., “I do not trust my memory”), (ii) Positive Beliefs about Worry (e.g., “Worrying helps me to solve problems”), (iii) Negative Beliefs about Uncontrollability and Danger (e.g., “My worrying thoughts persist no matter how I try to stop them”, “My worrying could make me go mad”), (iv) Cognitive Self-Consciousness (e.g., “I am constantly aware of my thinking”), and (v) Beliefs about the Need to Control Thoughts (e.g., “I should be in control of my thoughts all of the time”). Items are rated on how much the person generally agrees on a four-point scale ranging from 1 (Do not agree) to 4 (Agree very much). Higher scores indicate greater dysfunction in metacognition. The MCQ-30 demonstrates good construct validity, internal consistency and good test–retest reliability in non-clinical samples (Wells and Cartwright-Hatton, 2004; Spada et al., 2008; Fergus and Bardeen, 2019), cancer and epilepsy (Cook et al., 2014; Fisher et al., 2016), and psychiatric samples (Martin et al., 2014; Grötte et al., 2016).

#### Hospital Anxiety and Depression Scale (HADS; Zigmond and Snaith, 1983)

The HADS is a widely used self-report measure to assess anxiety and depression, each assessed by seven items. Items are rated based on the past 7 days on a four-point scale ranging from 0 to 3. The HADS yields two subscale scores and a total score. Subscale scores from 0 to 7 are categorized as normal, from 8 to 10 mild, from 11 to 14 moderate, and from 15 to 21 severe (Zigmond and Snaith, 1983). For the current study, all participants should have scored 8 or more on either of the subscales. The HADS is routinely used in cardiac rehabilitation services in the UK (National Audit of Cardiac Rehabilitation, 2017). The alpha values for the present sample were 0.81 for anxiety, and 0.76 for depression.

### Statistical Analyses

#### Descriptive Statistics

Descriptive statistics include means and standard deviations for the MCQ-30 and the HADS for the total sample and males and females separately; score distributions for the individual MCQ-30 items along with frequencies of missing responses; inter-correlations between the MCQ-30 and HADS; and Cronbach alpha values as measures of internal consistency. Independent sample *t*-tests were used to explore gender differences in the MCQ-30 and HADS.

#### Measurement Models

**FIGURE 1:**
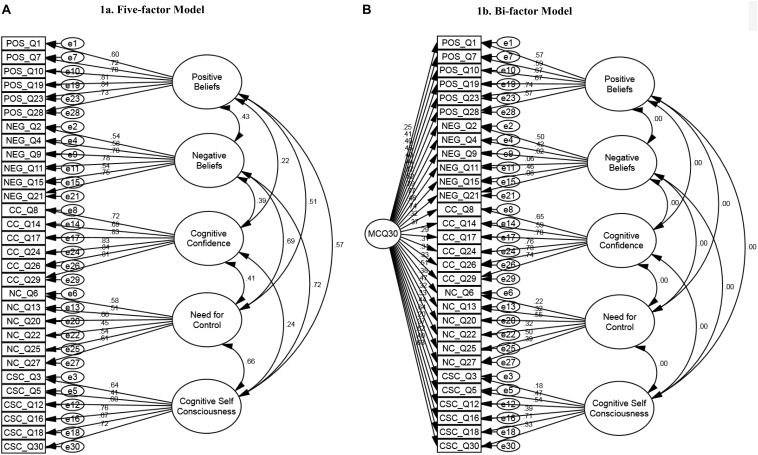
MCQ-30 five-factor and bi-factor models: Standardized factor loadings (regression weights). **(A)** Five-factor model. **(B)** Bi-factor model.

The factor structure of the MCQ-30 was investigated using confirmatory factor analysis (CFA). A unidimensional model was fitted first, principally to provide a baseline for comparison of the more complex models as the expectation was that this model would not fit the data well. Then, the two pre-specified models for the factor structure of the MCQ-30 were examined: (1) a five-factor model (Figure 1A) and (2) a bi-factor model (Figure 1B). Under the five-factor model each individual factor was allowed to freely correlate with all of the other factors; under the bi-factor model these correlations were constrained to be zero, as it is assumed that the general factor accounts for any relationships between sub-factors (Brown, 2015). Exploratory factor analysis using principal component analysis was conducted to examine alternative possible solutions and we tested if these provided a better fit to the data. As previous research showed that MCQ dimensions are intercorrelated, oblique (direct oblimin) rotation was computed.

#### Model Estimation and Evaluation

Confirmatory Factor Analysis (CFA) using maximum likelihood (ML) estimation was applied to examine the fit of the models to the MCQ-30 data. The MCQ-30 item scores demonstrated considerable skew, for which the ML approach has been shown to outperform other methods in overall fit and parameter estimates, including when data is ordinal (Olsson et al., 2000). The current sample of 440 is considerably larger than the minimum of 200 recommended for CFA (Kline, 2016). The statistical package used was AMOS Version 24 (Arbuckle, 2016).

The adequacy and parsimony of the models was principally assessed based on two statistical indices that are least sensitive to sample size and parameter estimates (Hu and Bentler, 1998): the Comparative Fit Index (CFI) and the Root Mean Square Error of Approximation (RMSEA) along with its 90% confidence interval. A CFI of 0.90 or above is commonly taken to indicate an acceptable fit (Kline, 2016), and previous studies of the MCQ-30 have all used this criterion value (e.g., Wells and Cartwright-Hatton, 2004; Grötte et al., 2016). However, we note that Hu and Bentler (1999) have argued for a more stringent level of 0.95. For comparability with previous studies of the MCQ-30, in this study we use 0.90 but regard that as representing minimum acceptability, with higher values being preferred. For the RMSEA, values >0.08 indicate an acceptable fit and 0.05 a good fit, with an upper 90% confidence limit of 0.1 or less (Browne and Cudeck, 1993). To provide a broader picture of model performance we also computed a number of secondary indices: the Goodness of Fit Index (GFI), with values closer to 1 indicating good fit; the Parsimony Goodness of Fit Index (PGFI), for which values above 0.5 indicate good fit (Hu and Bentler, 1999); and -for comparison with previous studies that have reported this index- the Tucker-Lewis Fit Index (TLI), for which 0.90 is taken to represent a good fitting model (Garver and Mentzer, 1999), and 0.95 following Hu and Bentler’s criterion (1999). We also report the Chi-square statistic, but goodness-of-fit decisions were not based on this criterion because this index is very sensitive to sample size and to high correlations between factors within the model, making it inappropriate for identifying well-fitting models (Tanaka, 1987). For all other indexes 0.90 was considered adequate and 0.95 good (see Bentler and Bonett, 1980; Hu and Bentler, 1999; Kline, 2005).

Our analysis procedure began by fitting the pre-specified five-factor and bi-factor models. Then, exploratory factor analysis was used to explore if an alternative solution provided a better fit to the data.

#### Regression Analysis

Hierarchical regression analysis was conducted to assess which MCQ-30 subscales significantly predicted anxiety and depression, after controlling for age and gender. Assumptions of linearity, homoscedasticity, independence of residuals and the normality of distributed errors were examined to determine whether regression analyses were appropriate (Field, 2018).

## Results

### Sample Demographics and Descriptive Statistics

The sample consisted of 440 participants. Sample demographics are presented in Table 1 along with means and standard deviations for the HADS and the MCQ-30. Twenty-two individual responses were missing (0.11%). No individual had more than two missing responses; missing values were replaced with the participant mean across the completed items.

**TABLE 1:** Sample demographic characteristics, HADS and MCQ-30 scores (*N* = 440).

**Age**	
Mean (*SD*)	60.24 (10.76)
Range	27–87
**Gender**	
Males	288 (65.5%)
Females	152 (34.5%)
**Ethnicity**	
White	400 (90.8%)
Black	10 (2.3%)
Asian	22 (5.0%)
Mixed	1 (0.2%)
Other	5 (1.1%)
Do not wish to disclose	2 (0.5%)
**Marital status**	
Single	73 (16.6%)
Married	215 (48.9%)
Cohabiting	40 (9.1%)
Civil partnership	3 (0.7%)
Separated	21 (4.8%)
Divorced	56 (12.7%)
Widowed	31 (7.0%)
Do not wish to disclose	1 (0.2%)
**Highest qualification gained**	
None	96 (21.8%)
GCSE or equivalent	93 (21.1%)
A-level or equivalent	29 (6.6%)
Vocational qualification	65 (14.8%)
Diploma	80 (18.2%)
Degree	59 (13.4%)
Master’s degree or PhD	17 (3.9%)
**HADS**	**Mean (*SD*)**
Anxiety	10.32 (3.85)
Depression	8.20 (3.71)
Total	18.52 (6.43)
**MCQ-30**	
Positive Beliefs	10.68 (4.49)
Negative Beliefs	13.16 (4.65)
Cognitive Confidence	11.50 (5.05)
Need for Control	11.86 (3.97)
Cognitive Self-Consciousness	14.62 (4.36)
Total	61.81 (16.02)

*HADS, Hospital Anxiety and Depression Scale; MCQ-30, Metacognitions Questionnaire 30.*

Independent samples *t*-tests exploring gender differences on the HADS and the MCQ-30 scores were significant for (i) HADS-Anxiety: males (*M* = 9.81, *SD* = 3.85) and females (*M* = 11.29, *SD* = 3.67); *t*_(320)_ = –3.95, *p* ≤ 0.001; and (ii) MCQ-30 Negative Beliefs about Uncontrollability and Danger: males (*M* = 12.71, *SD* = 4.58) and females (*M* = 13.99, *SD* = 4.68); *t*_(301)_= –2.75, *p* = 0.006. The differences were non-significant for all the remaining variables.

### MCQ-30 Item-Level Distribution, Internal Consistency, and Correlations

The response distributions on each of the MCQ-30 items are given in Table 2. Mean values for items ranged from 1.24 (item 22) to 2.80 (item 5). Table 2 shows there was substantial skew on many items, with one item (item 22) rated at the lower extreme of the response scale by 83.5% of participants and another seven items likewise rated by 50% or more.

**TABLE 2:** MCQ-30 descriptive data per item: Mean (M), Standard Deviation (SD), frequency and percentages per rating-scale options and missing items.

	M (*SD*)	1	2	3	4	Missing responses
Item 1	1.82(0.96)	216(49.1%)	120(27.3%)	72(16.4%)	32(7.3%)	
Item 2	2.22(1.09)	146(33.2%)	126(28.6%)	93(21.1%)	75(17.0%)	
Item 3	2.78(0.93)	41(9.3%)	125(28.4%)	161(36.6%)	112(25.5%)	1(0.2%)
Item 4	2.04(1.08)	184(41.8%)	120(27.3%)	72(16.4%)	64(14.6%)	
Item 5	2.81(1.00)	49(11.1%)	120(27.3%)	134(30.5%)	135(30.7%)	2(0.2%)
Item 6	1.95(1.08)	212(48.2%)	95(21.6%)	78(17.7%)	55(12.5%)	
Item 7	1.86(.99)	212(48.2%)	114(25.9%)	76(17.3%)	37(8.4%)	1(0.2%)
Item 8	2.14(1.09)	162(36.8%)	127(28.9%)	77(17.5%)	74(16.8%)	
Item 9	2.43(1.05)	102(23.2%)	132(30.0%)	118(26.8%)	86(19.6%)	2(0.4%)
Item 10	1.90(1.01)	203(46.1%)	118(26.8%)	77(17.5%)	42(9.6%)	
Item 11	2.49(1.07)	96(21.8%)	131(29.8%)	114(25.9%)	98(22.3%)	1(0.2%)
Item 12	2.01(1.02)	174(39.6%)	139(31.6%)	74(16.8%)	52(11.8%)	1(0.2%)
Item 13	2.67(1.12)	88(20.0%)	105(23.9%)	109(24.8%)	137(31.1%)	1(0.2%)
Item 14	2.10(1.06)	163(37.1%)	136(30.9%)	77(17.5%)	64(14.6%)	
Item 15	1.72(1.00)	259(58.9%)	84(19.1%)	59(13.4%)	38(8.6%)	
Item 16	2.61(1.05)	75(17.1%)	136(30.9%)	115(26.1%)	114(25.9%)	
Item 17	1.99(1.04)	185(42.0%)	133(30.2%)	65(14.8%)	57(13.0%)	
Item 18	2.24(1.05)	133(30.2%)	135(30.7%)	103(23.4%)	68(15.5%)	1(0.2%)
Item 19	1.68(0.91)	248(56.4%)	112(25.5%)	51(11.6%)	28(6.4%)	1(0.2%)
Item 20	1.91(1.08)	224(50.9%)	88(20.0%)	71(16.1%)	56(12.7%)	1(0.2%)
Item 21	2.27(1.05)	131(29.8%)	131(29.8%)	108(24.6%)	70(15.9%)	
Item 22	1.24(0.61)	370(84.1%)	44(10.0%)	18(4.1%)	8(1.8%)	
Item 23	1.78(0.91)	214(48.6%)	135(30.7%)	64(14.5%)	27(6.1%)	
Item 24	1.80(1.00)	235(53.4%)	98(22.3%)	69(15.7%)	37(8.4%)	1(0.2%)
Item 25	2.05(1.08)	182(41.4%)	115(26.1%)	79(18.0%)	63(14.3%)	1(0.2%)
Item 26	1.78(1.01)	244(55.5%)	88(20.0%)	67(15.2%)	40(9.1%)	1(0.2%)
Item 27	2.05(1.04)	173(39.3%)	126(28.6%)	85(19.3%)	55(12.5%)	1(0.2%)
Item 28	1.63(0.89)	263(59.8%)	93(21.1%)	62(14.1%)	20(4.6%)	2(0.4%)
Item 29	1.69(0.92)	245(55.7%)	110(25.0%)	57(13.0%)	27(6.1%)	1(0.2%)
Item 30	2.18(1.06)	147(33.4%)	132(30.0%)	93(21.1%)	67(15.2%)	1(0.2%)
						

Inter-correlations between MCQ-30 sub-scale scores were all significant (Table 3), ranging from 0.20 to 0.56. Cronbach alpha values for the MCQ-30 subscales ranged from 0.73 to 0.91 (Table 3). Table 3 shows that most correlations between the MCQ-30 and the HADS were significant, ranging from 0.21 to 0.63, except for the correlations between HADS-Depression and positive metacognitive beliefs and cognitive self-consciousness.

**TABLE 3:** MCQ-30: Internal consistency, inter-correlations among the latent factors and correlations with the Hospital Anxiety and Depression Scale (*N* = 440).

	2.	3.	4.	5.	6.	7.	8.	9.	Alpha
1. MCQ-30 positive beliefs	0.34***	0.20***	0.40***	0.46***	0.67***	0.36***	0.08*ns*	0.26***	0.88
2. MCQ-30 negative beliefs	−	0.36***	0.56***	0.55***	0.79***	0.63***	0.34***	0.57***	0.83
3. MCQ-30 cognitive confidence	−	−	0.33***	0.19**	0.61***	0.24***	0.34***	0.33***	0.91
4. MCQ-30 need for control	−	−	−	0.50***	0.76***	0.35***	0.21***	0.33***	0.73
5. MCQ-30 cognitive self–consciousness	−	−	−	−	0.75***	0.40***	0.06*ns*	0.27***	0.81
6. MCQ-30 total	−	−	−	−	−	0.55***	0.29***	0.50***	0.91
7. HADS-anxiety	−	−	−	−	−	−	0.45***	0.86***	0.81
8. HADS-depression	−	−	−	−	−	−	−	0.84***	0.76
9. HADS-total	−	−	−	−	−	−	−	−	0.84

MCQ-30, Metacognitions Questionnaire 30; HADS, Hospital Anxiety and Depression Scale. ***Correlation is significant at p < 0.001 (2-tailed); **Correlation is significant at *p* < 0.01 (2-tailed); ^*ns*^Non-significant.

### Confirmatory Factor Analysis

Goodness-of-fit statistics for each model are presented in Table 4. As expected, the unidimensional model did not reach criteria for adequate fit. The standard five-factor model demonstrated acceptable fit according to the RMSEA (and its confidence interval) criteria, but the CFI at 0.892 was just below the acceptability threshold. Results for the secondary fit criteria were mixed, with an acceptable fit according to the PGFI but not the GFI or TLI. Results for the bi-factor model showed an acceptable fit by both CFI and RMSEA criteria (and its confidence interval). Fit was also acceptable according to the PGFI, but not GFI or TLI.

**TABLE 4:** Goodness-of-fit statistics for tested models.

Models	χ^2^	df	*p*	CFI	RMSEA 90% CI [LL-UL]	GFI	PGFI	TLI
Unidimensional model	3421.798	405	<0.001	0.499	0.130 [0.126–0.134]	0.541	0.471	0.462
Five-factor model	1045.90	395	<0.001	0.892	0.061 [0.057–0.066]	0.856	0.727	0.881
Bi-factor model	899.24	375	<0.001	0.913	0.056 [0.052–0.061]	0.880	0.709	0.899

Incremental measures of fit, including the CFI and TLI, may be underestimated if the RMSEA for a “null” model is <0.158 (Kenny, 2015). For the current data the RMSEA for the null (independence) model, which assumed no correlations between observed variables and did not constrain means, was 0.178. We used a chi-square difference test to compare the five-factor and bi-factor models, a permissible test because the former was nested within the latter, and found a statistically significant increase in fit (Chi-square difference = 146.7, df = 20, *p* < 0.001). Standardized factor loadings (regression weights) and inter-correlations for both models appear in Figure 1.

Exploratory factor analysis resulted in a six-factor solution based on eigenvalues and the scree plot. However, the fit indices only marginally improved on those for the five-factor solution and therefore we did not pursue this solution any further [*χ*^2^_(390)_= 1008.001, *p* < 0.001, CFI = 0.897; RMSEA = 0.060 (95% CI 0.056 to 0.065); PGFI = 0.725; GFI = 0.865, TLI = 0.885].

### MCQ-30 Regression Analyses

Assumptions of linearity, homoscedasticity, independence of residuals, and normally distributed errors were met for the regression analyses.

The results of the hierarchical regressions are displayed in Table 5. Inclusion of the MCQ-30 subscales accounted for an additional 38% of the variance in HADS-Anxiety, with Positive and Negative Beliefs being independent significant predictors; gender was also a significant predictor (Table 5A). When predicting HADS-Depression, the inclusion of the MCQ-30 domain-specific subscales was significant and accounted for an additional 18% of the variance, with Negative Beliefs, Cognitive Confidence and Cognitive Self-Consciousness being significant unique factors; age was also a significant predictor (Table 5B).

**TABLE 5:** MCQ-30 subscales predicting anxiety and depression, after controlling for age and gender.

(A) MCQ-30 subscales predicting symptoms of anxiety						

	Step 1			Step 2		
		
	*B*	95% CI	*p*	*B*	95% CI	*p*
Age	–0.06	[–0.09, –0.02]	0.001	–0.02	[–0.05, 0.01]	0.133
Gender (male)	1.59	[0.84, 2.33]	<0.0001	0.86	[0.26, 1.47]	**0.005**
*R*^2^; F; *p*-value	0.056; 13.07; <0.001					
MCQ-30 positive beliefs				0.14	[0.07, 0.22]	**<0.0001**
MCQ-30 negative beliefs				0.45	[0.37, 0.53]	**<0.0001**
MCQ-30 cognitive confidence				0.01	[–0.05, 0.07]	0.687
MCQ-30 need for control				–0.03	[–0.12, 0.06]	0.542
MCQ-30 cognitive self–consciousness				0.02	[–0.06, 0.11]	0.600
*R*^2^; *R*^2^ change; *F* for change in *R*^2^; *p-*value				0.431; 0.375; 56.93; **< 0.001**		

**(B) MCQ-30 subscales predicting symptoms of depression**						

Age	–0.04	[–0.08, –0.01]	0.009	–0.03	[–0.06, 0.001]	**0.056**
Gender (male)	0.73	[0.003, 1.46]	0.049	0.47	[–0.22, 1.16]	0.183
*R*^2^; *F*; *p*-value	0.022; 5.00; 0.007					
MCQ-30 positive beliefs				–0.01	[–0.10, 0.07]	0.752
MCQ-30 negative beliefs				0.24	[0.14, 0.33]	**<0.0001**
MCQ-30 cognitive confidence				0.18	[0.12, 0.25]	**<0.0001**
MCQ-30 need for control				0.05	[–0.05, 0.16]	0.321
MCQ-30 cognitive self–consciousness				–0.15	[–0.25, –0.06]	**0.002**
*R*^2^; *R*^2^ change; F for change in *R*^2^; *p*-value				0.198; 0.176; 18.99; **< 0.001**		

*Bold values represent a significant p-value.*

## Discussion

This is the first study to investigate the factorial structure of the MCQ-30 (Wells and Cartwright-Hatton, 2004), a measure to assess metacognitive beliefs, in a cardiac population with co-morbid symptoms of anxiety and/or depression. Results of the CFA for the five-factor model just failed to meet our minimum criterion of CFI ≥ 0.9 although it did meet the RMSEA < 0.08 criterion, whereas the bi-factor model met both of these, although both models met only the PGFI secondary criteria set.

For clinical use of the MCQ-30, the findings suggest that the originally published five-factor latent structure of the instrument may not be ideal for cardiac patients. However, further investigations using other cardiac patient samples would be required to confirm this conclusion. The bi-factor solution suggests that this model carries additional information beyond that conveyed by the five subscale scores alone. The resulting structure suggests an underlying construct that draws on all of the subscales but is defined most strongly by items relating to uncontrollability. The factor may represent an overall level of reduced flexibility in cognition (e.g., attentional control) that in metacognitive theory is hypothesized to be a common contributor to psychological vulnerability (Wells, 2019).

The bi-factor model is promising, however, only one other study using non-clinical sample has tested this solution (Fergus and Bardeen, 2019); and in our view the stability and usefulness of such a model needs to be assessed further before widespread adoption could be recommended. It is also computationally much more complex to derive scores from the bi-factor solution and their interpretation is not as simple. Considering also that all five subscales demonstrated good internal consistency in themselves and showed similar relationships to HADS scores as in other patient populations, for practicality we recommend continued use of the standard five-factor and its interpretation in cardiac patients, at least for the time-being. This conclusion is also supported by this solution exhibiting similar levels of fit in terms of CFI and RMSEA to the majority of previous metacognitive studies that have explored the factorial structure among both non-clinical and clinical populations.

The patient population in the current study was markedly different to those studied in previous research undertaken on the MCQ-30 five-factor structure, being characterized by mild to severe anxiety and/or depression symptoms in the context of a specific physical condition. Compared to studies conducted in non-clinical (Wells and Cartwright-Hatton, 2004; Spada et al., 2008; Ramos-Cejudo et al., 2013), and cancer and epilepsy populations (Cook et al., 2014; Fisher et al., 2016), participants in the present study exhibited higher levels of problematic metacognitive activity, with the highest or second-highest mean scores on all five MCQ-30 subscales.

The results support previous studies of positive relationships between metacognitive factors and anxiety and depression symptoms (Wells and Cartwright-Hatton, 2004; Spada et al., 2008; Yilmaz et al., 2008). In a recent meta-analysis (Sun et al., 2017) and in previous studies conducted in mental health, physical illness, student and community samples (e.g., Allott et al., 2005; Spada et al., 2008; Yilmaz et al., 2008), the MCQ-30 negative beliefs subscale concerning uncontrollability and danger was the stronger predictor of anxiety and depression. This result also holds in the current sample. Specifically, negative metacognitive beliefs of uncontrollability and danger positively accounted for variance in each distress subscale, but there were additional contributors in each case. In the case of anxiety, positive metacognitive beliefs also contributed; whilst in depression, cognitive confidence and cognitive self-consciousness made individual additional contributions. Interestingly, for the latter variable the relationship was negative, suggesting that lower cognitive self-consciousness was individually associated with greater depression. Findings provide evidence for a trans-symptomatic metacognitive correlate, i.e., negative metacognitive beliefs of uncontrollability and danger, with some more specific additional contributions that further explain the role of metacognitions on anxiety and depression in cardiac patients.

### Strengths and Limitations

The strengths of this study include a sample of over 400 participants used to test factorial models of the MCQ-30. Furthermore, the amount of missing data was very small (0.11%); ensuring information for all the variables included in the analysis was reliable. However, study limitations must be acknowledged. First, self-report measures were used, and this may have introduced self-report bias. Second, data were not collected to examine test-retest reliability of the MCQ-30 in this sample because the PATHWAY-Programme involved the delivery of a psychological intervention. Third, whilst the bi-factor model met the minimum criteria for goodness of fit, the more stringent Hu and Bentler’s criterion (1999) was not satisfied.

## Conclusion

In summary, CFA analysis suggests that the originally published latent structure of five-factors of the MCQ-30 may not be generalizable to distressed cardiac patients. The bi-factor model had a better fit and should be investigated in future studies. Nevertheless, the current data confirmed that individual dimensions of metacognition explain anxiety and depression symptoms among cardiac patients, supporting an extension of metacognitive theory and therapy of psychological distress to this group of patients.

## Data Availability Statement

The datasets generated for this study are available on request to the corresponding author.

## Ethics Statement

Ethical approval for the PATHWAY programme has been granted by the NHS Research Ethics Committee, United Kingdom (References: 15/NW/0136 and 16/NW/0786). All patients provided written informed consent.

## Author Contributions

AW was the co-developer of the MCQ-30. AW, CF, and DR designed the study. CF contributed to the recruitment, consent, administration of the baseline measures to participants, and drafted the initial manuscript. CF and CH conducted the statistical analysis supervised by DR and AW. DR and AW revised the manuscript. All authors contributed and agreed the final draft.

## Conflict of Interest

AW was the developer of metacognitive therapy, co-developer of the MCQ-30 and the director of the Metacognitive Therapy Institute. The remaining authors declare that the research was conducted in the absence of any commercial or financial relationships that could be construed as a potential conflict of interest.
